# Rupture utérine spontanée en livre ouvert sur grossesse de 15 semaines chez une paucipare avec utérus cicatriciel: à propos d′un cas

**DOI:** 10.11604/pamj.2020.36.44.20692

**Published:** 2020-05-28

**Authors:** Eloge Ilunga-Mbaya, Olivier Nyakio, Raha Maroyi, Patrick Bigabwa, Moise Kiminyi, Silas Hamisi, Dénis Mukwege, Dieudonné Sengeyi Mushengezi Amani

**Affiliations:** 1 Département de Gynécologie et Obstétrique, Cliniques Universitaires de Kinshasa, Faculté de Médecine de l′Université de Kinshasa, Kinshasa, République Démocratique du Congo; 2 Département de Gynécologie et Obstétrique, Hôpital Général de Référence de Panzi, Faculté de Médecine de l’Université Evangélique d’Afrique, Bukavu, République Démocratique du Congo

**Keywords:** Rupture utérine, deuxième trimestre, utérus cicatriciel, Uterine rupture, second trimester, scarred uterus

## Abstract

Malgré toutes les politiques sanitaires mises en œuvre dans nos pays en développement, les ruptures utérines (RU) restent fréquentes. Elles revêtent des formes graves mettant en jeu le pronostic maternel et fœtal. Parmi les nombreux facteurs de risque, le plus fréquent cité par tous les auteurs est la cicatrice de césarienne. La plupart surviennent durant le travail ou en fin de grossesse. Les ruptures utérines au 1^e^ et 2^e^ trimestre restent exceptionnelles et leur expression clinique variable. Les auteurs rapportent un cas de rupture utérine spontanée sur grossesse de 15 semaines survenue chez une paucipare avec antécédent d'utérus cicatriciel. La clinique a été marquée par un tableau d'irritation péritonéale. L'exploration chirurgicale a révélé une rupture complète verticale allant du fond utérin au segment inférieur et ouvrant l'utérus en livre ouvert. La RU devrait donc être envisagée de principe devant un tableau douloureux abdominal avec signes d'hémopéritoine chez une gestante avec utérus cicatriciel, quel que soit le terme et même dans les deux premiers trimestres de grossesse, quel que soit l'âge (jeunes patientes) et la parité.

## Abstract (en)

Despite all the health policies implemented in our developing countries, uterine rupture (RU) remains common. They are severe and involve maternal and fetal prognosis. Among the multiple risk factors, the most common cited in the literature is C-section scar. Most occurs during labor or at the end of pregnancy. Uterine rupture during the first or second trimester is exceptional and its clinical manifestation is variable. We here report a case of spontaneous uterine rupture at 15 weeks' gestation in a pauciparous woman with scarred uterus. Exacerbation of clinical symptoms was manifested by peritoneal irritation. Surgical exploration revealed complete vertical rupture from the bottom to the lower segment of the uterus with open book opening of the uterus. This study highlights that uterine rupture should be considered in patients with scarred uterus presenting with abdominal pain associated with signs of hemoperitoneum, regardless of whether their pregnancies are at term or in the first two trimesters, and regardless of age (young patients) and parity.

## Introduction

La rupture utérine (RU) qui est un des accidents les plus graves qui puisse survenir au cours du travail ou en fin de grossesse, contribue pour beaucoup dans la mortalité maternelle et fœtale dans les pays en développement [1]. Ses facteurs de risque sont les antécédents de césarienne ou des chirurgies gynécologiques (myomectomie), les malformations utérines, l'usage abusif des utérotoniques etc. La plupart surviennent généralement au cours du travail chez les gestantes ayant les facteurs de risque. Parmi ces ruptures utérines spontanées, seulement 17% apparaissent avant le début du travail [2, 3]. Cependant, son incidence au 1^e^ et 2^e^ trimestre de la grossesse est rare [2]. Lorsque la symptomatologie apparait tôt dans la grossesse, le diagnostic porté avant la laparotomie est celui d'avortement tardif [3]. Lorsqu'une échographie est faite soit elle est normale et le diagnostic reste incertain, soit elle montre l'hémopéritoine et alors la discussion se fait entre l'hémorragie obstétricale ou une hémorragie chirurgicale (rupture splénique spontanée, rupture vasculaire d'autres origines) [3]. En début du 2^e^ trimestre, une symptomatologie faite des douleurs abdominales, vomissements, fièvre et arrêt des matières peut prêter à confusion. Devant ce réel problème diagnostique, nous rapportons un cas de rupture utérine en livre ouvert sur grossesse de 15 semaines chez une gestante avec antécédents d'utérus cicatriciel.

## Patient et observation

**Figure 1:**
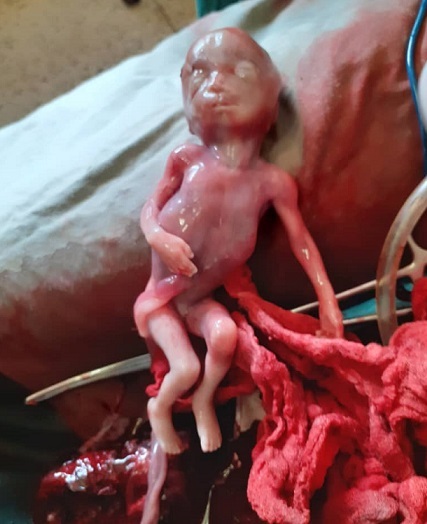
Fœtus accompagné des annexes

**Figure 2:**
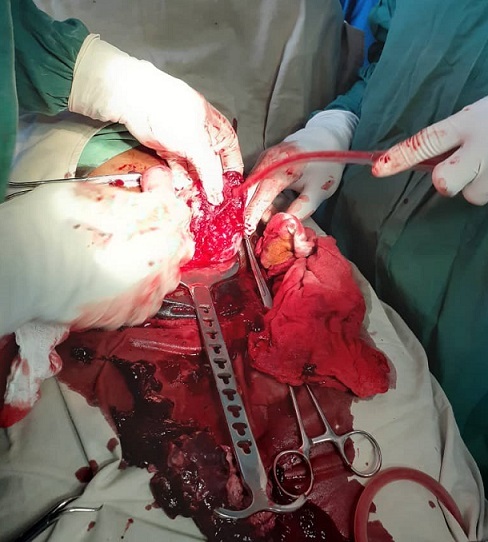
Utérus en livre ouvert

Il s'agit de madame N, âgée de 25 ans, 2^e^ pare et 3^e^ geste. Transférée d'une polyclinique située à 30 km de la ville de Bukavu pour abdomen aigu chirurgical sur aménorrhée de 15 semaines selon la note de transfert. Ses antécédents sont essentiellement marqués par deux césariennes, la première en 2009 pour bassin rétréci et la 2^e^ itérative première en 2015. Les comptes rendus opératoires de ces césariennes n'ont pas été disponibilisés. Les nouveau-nés qui ont respectivement pesé 2300 et 2500 grammes sont en vie. Elle n'avait pas encore débuté les consultations prénatales (CPN) et n'avait réalisé aucune échographie. Sa date des dernières règles remontait au mois d'avril (sans autres précisions) soit environ 3 mois au moment de l'admission. Son histoire rapportait le début à une semaine dans un tableau de menace d'avortement tardif, soigné comme faisant le paludisme dans un dispensaire. La persistance des hypogastralgies motivera son transfert à la polyclinique sus indiquée où elle restera 24 heures avec un traitement fait de Ceftriaxone, Gentamicine, Métronidazole et l'évolution sera marquée par des fièvres, douleurs abdominales diffuses, vomissements et arrêt des matières, ce qui motivera son transfert à la maternité de l'Hôpital Général de Référence de Panzi. Elle ne présentait aucune métrorragie. A son admission, la tension artérielle était de 95/54 mmHg, pouls à 113 pulsations/minute, la fréquence respiratoire à 25 cycles/minute, la température à 38ºC et une taille de 148 cm pour 50 kg. Elle était agitée et peu colorée. A l'inspection, l'abdomen ne semblait pas ballonné. Les manœuvres de Léopold étaient difficiles à réaliser à cause d'une sensibilité abdominale diffuse avec un signe de rebound positif. Le col était postérieur, long et fermé. On palpait en postérieur une masse comblant le rectum. Au toucher rectal (TR) on avait un fécalome haut situé comblant la lumière et difficile à accoucher. L'échographie réalisée révéla un fœtus de 15 semaines sans activité cardiaque. Un placenta bas situé type III, un épanchement péritonéal plus ou moins abondant, une dilatation des anses intestinales et du liquide de stase faisant évoquer une péritonite. La numération formule sanguine réalisée montra une hémoglobine à 9,6 mg/dl, hématocrite à 27,9 gr%, les leucocytes à 34 000 éléments/mm^3^ avec formule leucocytaire à prédominance neutrophilique, plaquettes à 344 000 éléments/mm^3^, le groupe sanguin O Rhésus positif. La fonction rénale était normale ainsi que l'ionogramme sanguin. Devant ce tableau, il fut décidé de réaliser une laparotomie exploratrice pour suspicion d'une péritonite sur grossesse et l'éventualité d'un hémopéritoine sur probable déhiscence utérine n'était pas exclue. L'équipe de chirurgie générale a été associée à la prise en charge du cas et l'intervention fut réalisée sous anesthésie générale. Après la coeliotomie médiane nous avons noté un hémopéritoine d'environ 500 cc, le fœtus et ses annexes baignant dans la cavité abdominale et une rupture utérine verticale allant du fundus au segment inférieur d'environ 8 centimètres de long et ouvrant l'utérus en livre. Nous avons réalisé alors un traitement conservateur en pratiquant une hystérorraphie suivie du nettoyage de la cavité abdominale avec 4 litres de sérum physiologique tiède et la pose d'un drain dans le cul-de-sac de Douglas. L'exploration de l'étage sus mésocolique n'a rien noté de particulier. Un test au bleu de méthylène pour s'assurer de l'étanchéité vésicale a été réalisé. La patiente a bénéficié d'une transfusion et le fœtus a pesé 309 gr. Les suites opératoires étaient simples et un compte rendu opératoire lui a été remis (Figure 1, Figure 2).

## Discussion

La rupture utérine qui reste une préoccupation de l′obstétricien en Afrique subsaharienne est définie comme toute solution de continuité non chirurgicale de la paroi utérine survenant pendant la grossesse ou lors du travail [4, 5]. Il est classique de distinguer d'une part la rupture utérine complète se caractérisant par une rupture totale de la paroi utérine mettant en communication la cavité utérine et le péritoine (les membranes pouvant être intactes ou pas) et d'autre part la rupture utérine incomplète (déhiscence) qui se caractérise par une rupture de l'endomètre et myomètre sans atteinte du péritoine viscéral [6]. Son incidence est faible dans les pays industrialisés (0,2 - 0,8%) [7, 8], pourtant dans nos pays en développement malgré toutes les pratiques sanitaires mises en œuvre, les ruptures utérines demeurent fréquentes [9]. Bien que la rupture utérine sur utérus sain soit décrite, la cicatrice utérine est au premier plan des facteurs de risque, ainsi que la grande multiparité [6,10]. Le type d'incision utérine est le principal facteur majorant le risque de rupture utérine, le risque étant plus élevé en cas d'incision corporéale contrairement à une incision segmentaire transversale basse [10, 11]. La rupture utérine peut se produire à tout âge gestationnel mais elle survient dans 75 à 80% des cas au cours du travail lors d'une tentative d'accouchement par voie basse après césarienne et dans la 2^e^ moitié de la grossesse [5, 12]. Une rupture utérine sur grossesse jeune en début du 2^e^ trimestre reste donc exceptionnelle dans la littérature. Nous nous intéressons donc à ce type de rupture. La rupture utérine précoce (au cours du 2^e^ trimestre) survient dans des situations particulières (grossesse cornuale ou interstitielle, antécédent de perforation, grossesse sur cicatrice de césarienne etc.), néanmoins le faible nombre d'observations collectées ne permettrait pas de définir des facteurs de risques fiables [13]. Le plus fréquent des facteurs prédisposant reconnu par tous les auteurs est la cicatrice utérine après césarienne. N'ayant pas de compte rendu opératoire des césariennes antérieures du cas en question, une cicatrice corporéale antérieure pourrait donc expliquer cette survenue précoce. Ses manifestations cliniques sont variables, le diagnostic est le plus souvent évoqué en cours de travail devant un faisceau d'arguments parmi lesquels des anomalies du rythme cardiaque fœtal (RCF), douleurs abdominales d'apparition secondaire, hémorragie génitale, hématurie macroscopique et des modifications de la dynamique utérine [12, 14]. Cependant, sur des grossesses jeunes telles que présentées ici, sa symptomatologie reste variable. La survenue des métrorragies serait en revanche moins fréquente mais les signes d'hémopéritoine quasi constant [15, 16].

L'échographie abdomino-pelvienne a comme intérêt principal de mettre en évidence l′hémopéritoine et le diagnostic ne sera finalement confirmé qu'en per opératoire lors de la laparotomie [10]. Bien que dans la série de Diallo [9], 88% de rupture utérine siège sur le segment inférieur, Voogd [17] et Margulies [18] signalent que lorsque la rupture apparait avant le travail elle concerne le corps et pendant le travail le segment inférieur. Sur ce, les modalités de la réparation chirurgicale (hystérectomie ou traitement conservateur) dépendent des lésions anatomiques constatées, de l'âge, la parité, le niveau socio-économique, l'ethnie, l'expérience de l'opérateur etc. Jusque dans les années 80, l'hystérectomie était le traitement préconisé dans les ruptures utérines complètes et en l′absence de l'hystérectomie une ligature des trompes était pratiquée [7]. Des séries des cas de grossesse après traitement conservateur d'une RU et poursuivies sans récidive remettent en question les dogmes établis [19]. Pour O′Connor *et al.* [20], un traitement conservateur avec réparation des lésions utérines doit être entrepris lorsqu'il est techniquement possible. Une grossesse ultérieure n'est pas contre indiquée en cas de RU mais le risque de récurrence (de l'ordre de 4-19%) augmente surtout en cas de cicatrice corporéale [10]. Pour ce qui est du cas rapporté on se retrouve devant une RU spontanée en début du 2^e^ trimestre chez une gestante paucipare avec antécédent de 2 césariennes. A notre avis, des cicatrices corporéales antérieures et le segment inférieur non encore formé pourraient respectivement expliquer cette précocité et le siège de la rupture. Bien que les douleurs abdominales et les vomissements soient parmi les signes classiques de RU, il était difficile d′y pensé étant donné la précocité et le caractère non spécifique de ces symptômes pouvant aussi être constatés en cas de péritonite et autres syndromes. L'échographie a eu un rôle limité à l'identification d'un épanchement péritonéal et finalement le diagnostic n'a été posé qu'après la laparotomie. Devant le jeune âge de la patiente, le désir de maternité, un traitement conservateur a donc été entrepris bien que le risque de récidive à la prochaine grossesse soit de l'ordre de 4 à 19% [10].

## Conclusion

La rupture utérine qui est devenue exceptionnelle et presque nulle ailleurs reste préoccupante pour nous. Un diagnostic précoce suivi d'une prise en charge rapide est essentiel pour réduire la mortalité materno fœtale. Au vu de ce qui précède, il parait donc important d'évoquer le principe de la rupture utérine devant un tableau douloureux abdominal avec signes d'hémopéritoine chez une gestante avec utérus cicatriciel, quels que soit le terme et même dans les deux premiers trimestres de grossesse, quel que soit l'âge (jeunes patientes) et la parité.

## Conflits d’intérêts

Les auteurs ne déclarent aucun conflit d′intérêts.

## References

[1] Nyengidiki TK, Allagoa DO (2011). Rupture of the gravid uterus in a tertiary health facility in the Niger Delta region of Nigeria: a 5 year review. Niger Med J.

[2] Abbas AM, Reda SH, Mustafa NA (2018). Spontaneous first trimester posterior uterine rupture in a multiparous woman with scarred uterus: A case report. Middle East fertility society journal.

[3] Bretones S, Cousin C, Gualandi M, Mellier G (1997). Rupture utérine. A propos d'un cas de rupture spontanée à 30 SA chez une primipare. J Gynecol Obstet Biol Reprod (Paris).

[4] De Tourris H, Henrion R, Delecour M (1994). Abrégé illustré de Gynécologie Obstétrique. Masson.

[5] Hofmeyr GJ, Say L, Gülmezoglu AM (2005). Systematic Review: WHO systematic review of maternal mortality and morbidity: the prevalence of uterine rupture. BJOG.

[6] Sentilhes L, Vayssière C, Beucher G, Deneux-Tharaux C, Deruelle P, Diemunsch P (2013). Delivery for women with a previous cesarean: guidelines for clinical practice from the French College of Gynecologists and Obstetricians (CNGOF). Eur J Obstet Gynecol Reprod Biol.

[7] Eden RD, Parker RT, Gall SA (1986). Rupture of the pregnant uterus: a 53-year review. Obstet gynecol.

[8] Gautier G, Vanbell Y, Vanbogaert LJ (1985). Rupture utérine: réflexion à propos d'un cas spontané à mi grossesse. J Gynecol Obstet Biol Reprod.

[9] Diallo FB, Idi N, Vangeenderhuysen, Baraka D, Hadiza I, Garba M (1998). La rupture utérine à la maternité centrale de référence de Niamey: aspects épidémiologiques et stratégies de prévention. Dakar Med.

[10] Parant O (2012). Rupture utérine:prédiction, diagnostic et prise en charge. J Gynecol Obstet Biol Reprod (Paris).

[11] Kayem G, Raiffort C, Legardeur H, Gavard L, Mandelbrot L, Girard G (2012). Critères d'acceptation de la voie vaginale selon les caractéristiques de la cicatrice utérine. J Gynecol Obstet Biol Reprod (Paris).

[12] Fitzpatrick KE, Kurinczuk JJ, Alfirevic Z, Spark P, Brocklehurst P, Knight M (2012). Uterine rupture by intended mode of delivery in the UK: a national case control study. PLoS Med.

[13] Cayrac M, Faillie JL, Flandrin A, Boulot P (2011). Second- and third-trimester management of medical termination of pregnancy and fetal death in utero after prior caesarean section. Eur J Obstet Gynecol Reprod Biol.

[14] Barger MK, Weiss J, Nannini A, Werler M, Heeren T (2011). Stubblefield PG. Risk factors uterine rupture among women who attempt a vaginal birth after a previous cesarean: a case control study. J Reprod Med.

[15] Mozurkewich EL, Hutton EK (2000). Elective repeat cesarean delivery versus trial of labor: a meta-analysis of the literature from 1989 to 1999. Am J Obstet Gynecol.

[16] Dewane JC, Mac Cubbin JH (1981). Spontaneous rupture of an unscarred uterus at 19 weeks gestation. Am J Obstet Gynecol.

[17] Voogd LB, Wood HB, Powell DV (1956). Ruptured uterus. Obstet Gynecol.

[18] Margulies D, Crampazona JT (1966). Rupture in the intact uterus. Obstet Gynecol.

[19] Chibber R, El-Saleh E, Al Fadhli R, Al Jassar W, Al Harmi J (2010). Uterine rupture and subsequent pregnancy outcome-how safe is it? A 25-year study. J Matern Fetal Neonatal Med.

[20] O'Connor RA, Gaughan B (1989). Pregnancy following simple repair of the ruptured gravid uterus. Br J Obstet Gynaecol.

